# The Impact of Regional Nerve Blocks on Postoperative Delirium or Cognitive Dysfunction following Thoracic Surgery: A Systematic Review and Meta-Analysis

**DOI:** 10.3390/jcm12247576

**Published:** 2023-12-08

**Authors:** Su Yeon Kim, Jiyoun Lee, Hyo-Seok Na, Bon-Wook Koo, Keum O Lee, Hyun-Jung Shin

**Affiliations:** Department of Anesthesiology and Pain Medicine, Seoul National University Bundang Hospital, Seoul 13620, Republic of Korea; kittyrockz@nate.com (S.Y.K.); 89726@snubh.org (J.L.); hsknana@gmail.com (H.-S.N.); bwkoo@snubh.org (B.-W.K.); lko0861@hotmail.com (K.O.L.)

**Keywords:** delirium, nerve block, postoperative cognitive dysfunction

## Abstract

Regional nerve blocks (NBs) mitigate the occurrence of postoperative cognitive dysfunction (POCD) and postoperative delirium (POD) in adult patients undergoing thoracic surgery. This study aimed to determine the exact effect of NBs on POCD and POD. Electronic databases, including PubMed, EMBASE, CINAHL, Scopus, and Web of Science, were searched for studies. The primary outcome was the incidence of POD or POCD. The secondary outcome was pain scores assessed 24 and 48 h postoperatively. We calculated the log odds ratio (LOR) and standardized mean difference (SMD) with 95% confidence intervals (CIs). The LOR was converted to an odds ratio (OR). In the analysis of 1010 patients from seven randomized controlled trials, POD and POCD rates were 14.1% and 16.7%, respectively, in the NB group, and higher, at 27.3% and 35.2%, in the control group. NBs reduced the incidence of POD (OR, 0.44; 95%CI 0.30 to 0.64; *p* < 0.001; I^2^ = 0.00%) and POCD (OR, 0.43; 95%CI 0.24 to 0.76; *p* < 0.001; I^2^ = 0.00%). NBs reduced pain scores at 24 h (SMD, −2.60; 95%CI −3.90 to −1.30, *p* < 0.001; I^2^ = 97.68%) and 48 h (SMD, −1.80; 95%CI −3.18 to −0.41, *p* = 0.01; I^2^ = 98.14%) postoperatively. NBs mitigated the occurrence of POD and POCD in adult patients after thoracic surgery.

## 1. Introduction

Impairment of cognitive function is one of the adverse outcomes of surgery [1]. Postoperative delirium (POD) and postoperative cognitive dysfunction (POCD) emerge as the most frequent symptoms of cognitive change during the recovery period from surgery [1]. These alterations in cognitive capacity can result in an increased length of hospital stay, extended length of intensive care unit stay, increased incidence of adverse medical events, elevated probabilities of hospital readmission, and augmented mortality rates [2,3].

Several etiologies are recognized as risk factors for POD or POCD, such as age, elevated American Society of Anesthesiologists score, pre-existing cognitive impairment, and psychiatric disorders [4]. Moreover, inadequately controlled postoperative pain could also significantly influence the occurrence of postoperative neurocognitive disorders [1,5]. Vaurio et al. [6] reported that older adults undergoing major non-cardiac surgery are susceptible to POD development, particularly when experiencing moderate-to-severe preoperative pain and a substantial increase in pain.

Modern multimodal analgesic techniques recommend the use of regional nerve blocks, either with a peripheral or neuraxial approach, to optimize perioperative pain management. Regional nerve blocks are applied to surgical patients for postoperative pain control [7]. Recently, several clinical trials have reported conflicting results regarding the effect of regional nerve blocks on the occurrence of POD or POCD [8,9].

Various nerve blocks can be performed on patients undergoing thoracic surgery. Some of the prominent ones include intercostal nerve block, serratus anterior plane block, thoracic paravertebral block, and erector spinae plane block [10,11,12]. In patients undergoing thoracic surgery, the incidence of postoperative delirium tends to be relatively high, reported at approximately 28% [13]. Various attempts have been made to reduce such postoperative delirium, including the use of dexmedetomidine [14], non-steroidal anti-inflammatory drugs [15], melatonin [16], ketamine [17], and non-pharmacological strategies such as early mobilization or reorientation [18]. In surgeries other than thoracic procedures, nerve block has been demonstrated to contribute to reducing the incidence of postoperative delirium [19,20].

This systematic review and meta-analysis aimed to delineate the advantages of regional nerve blocks, specifically assessing the effects of nerve blocks on postoperative cognitive function among patients after thoracic surgery.

## 2. Methods

This systematic review and meta-analysis adhered to the guidelines outlined in the Preferred Reporting Items for Systematic reviews and Meta-analyses statement [21] for conducting and reporting RCT-based systematic reviews. The study protocol was duly registered in the International Prospective Register of Systematic Reviews (PROSPERO, identifier: CRD42022310839).

### 2.1. Eligible Criteria

The following inclusion criteria were applied to select the relevant literature: (P) patients undergoing thoracic surgery via thoracoscopic or thoracotomy; (I) regional nerve block; (C) no block; and (O) evaluation of the incidence of POD or POCD. We excluded studies from the present analysis if they were not RCTs (e.g., observational study, retrospective study, or review article); if the full text, including the abstract or protocol, was not available; if regional nerve block was not performed; or if participants did not undergo thoracic surgery.

### 2.2. Search Strategy

We sought eligible trials by searching electronic databases, such as PubMed, EMBASE, SCOPUS, CINAHL, and Web of Science, covering the period from inception to 11 January 2022. No limitations were applied regarding publication year, language, journal, or region. The terms used for search consisted of “block”, “nerve block”, “delirium”, “cognitive dysfunction”, “cognitive impairment”, “thoracic”, or “thoracotomy”. The comprehensive strategy, along with the limits, is outlined in Supplementary Materials Table S1.

### 2.3. Study Selection

Two separate reviewers (SYK and JL) executed the study selection process following the inclusion criteria. Upon amalgamating the studies, initial screening was conducted on the basis of the title and abstract to identify pertinent studies. Subsequently, a thorough assessment of the full text was conducted for the selected studies. Inclusion criteria guided the final selection. Any discrepancies between the two reviewers were resolved through discussion with a third reviewer (HJS).

### 2.4. Data Extraction

Two independent reviewers (SYK and JL) extracted outcome data from the final randomized controlled trials (RCTs) identified during the study selection process. The extracted variables, including authors, publication year, number and age of participants, type of surgery, specific nerve block performed, local anesthetic utilized, and pain scores assessed after surgery, were collated into spreadsheets for summary. We applied Wan’s formula to estimate the mean and standard deviations for continuous data presented as median with interquartile [22]. We used WebPlotDigitizer (https://apps.automeris.io/wpd/; accessed on 1 March 2022) to convert graphical data into numerical values.

### 2.5. Assessment of the Risk of Bias

Using the Cochrane risk of bias tool for randomized trials [23], two reviewers independently assessed the risk of bias. Seven categories determined random sequence generation, allocation concealment, blinding of participants, blinding of outcome collectors, incomplete outcome data, selective reporting, and other potential biases. Each risk of bias was classified into “low risk”, “unclear”, or “high risk”, and the reasons regarding the assignment are summarized in Table S2.

### 2.6. Outcome Measures

The incidence of POD and POCD was the primary outcome. Postoperative pain scores, which were assessed using an 11-point visual analog scale or numerical rating scale (NRS), where 0 signified no pain and 10 represented extreme pain, in the first postoperative 24 h were the secondary outcome of the present study.

### 2.7. Statistical Analysis

Data analysis was performed using Stata SE version 17 (Stata Corp., College Station, TX, USA). We computed the log odds ratio (LOR) to compare effect sizes for the primary outcomes, which were dichotomous variables. When discussing the incidence of POD and POCD, the LOR was transformed into OR. Pain scores (secondary outcomes) represented continuous numerical data, and standardized mean differences (SMD) were computed using Hedges’ g statistic. A random-effects model was chosen for analysis because of the likelihood that different studies would reflect different effect sizes based on their methods and samples.

A sensitivity analysis was conducted by omitting studies to assess whether any single study could skew the pooled effect sizes. We assessed the heterogeneity in pooled effect estimates using the Cochran Q test and I^2^ statistic, categorizing the levels of heterogeneity as high (I^2^ = 76–100%), moderate (I^2^ = 26–75%), or low (I^2^ = 0–25%). Publication bias was evaluated through the funnel plot, Egger’s linear regression test, and the “trim and fill” procedure. *p* < 0.05 was considered statistically significant.

## 3. Results

### 3.1. Study Selection

**Figure 1:**
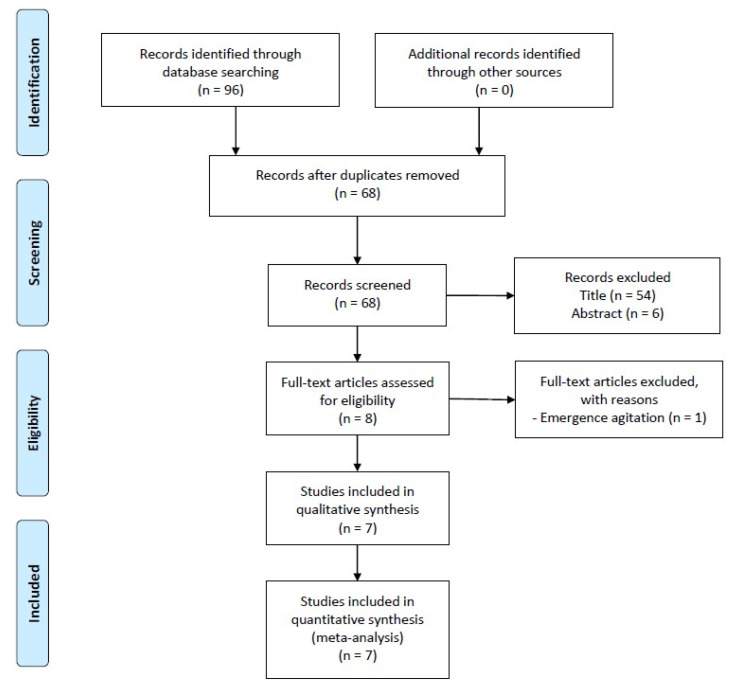
Preferred Reporting Items for Systematic Reviews and Meta-analyses flow diagram of study selection. A total of 96 articles were identified from the electronic databases. After excluding 28 studies due to duplication, 54 and 6 articles were removed from the article pool based on the title and abstract, respectively. Then, the full texts of the eight eligible studies were reviewed, and one study was excluded from the final analysis due to the inconsistency in the measured outcome. Finally, seven RCTs were chosen for the final analysis.

Ninety-six articles were initially found in the electronic database. After removing 28 studies as duplicates, 54 articles were removed based on the title, and an additional 6 were excluded after reviewing their abstracts. Subsequently, the full texts of the eight eligible studies were assessed, leading to the exclusion of one study [24] from the final analysis due to inconsistency in the measured outcome. Seven RCTs were selected for the final analysis [8,9,25,26,27,28,29] (Figure 1). Table 1 presents the characteristics of each RCT. A total of 1010 participants were included, and 504 and 506 participants were allocated to the intervention and control groups, respectively. The details regarding the performed nerve block procedures in each included trial are described in Table 2.

A study by Xie et al. [28] consisted of three groups: an ultrasound-guided thoracic paravertebral block (TPVB) group, epidural block (EB) group, and control group. In order to delineate the impacts of the TPVB and EB groups, we treated Xie et al.’s study as comprising two separate analyses. Statistical comparisons were conducted between the TPVB and EB groups individually against the control group. The TPVB group was denoted as Xie_1, while the EB group was designated as Xie_2.

Five studies [8,9,25,26,28] performed TPVB, two studies [27,28] performed EB, and one study [29] performed intercostal nerve block (ICNB). Three main methods were used to diagnose POD or POCD in included studies: the mini-mental state examination (MMSE) [27,28,29], confusion assessment tool (CAM) [8,9,26], and nursing delirium screening scale (NDSS) [25]. Participants in four studies [8,25,27,28] underwent video-assisted thoracic surgery (VATS), while those in three other studies received thoracotomy either for esophageal cancer [26,29] or transapical aortic valve replacement [9].

### 3.2. Postoperative Delirium

**Figure 2:**
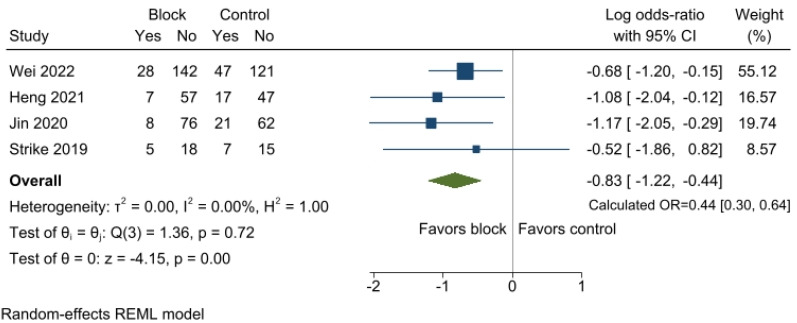
Forest plot of the incidence of POD between the regional nerve block and control groups. Participants who received regional nerve block showed notable differences in the incidence of POD compared to those who did not receive the block. POD, postoperative delirium; OR, odds ratio; CI, confidence interval [8,9,25,26].

Four studies reported the incidence of POD, and data were pooled from 678 participants [8,9,25,26]. The incidence of POD was 14.1% (48/341) and 27.5% (92/337) in the regional block and control groups, respectively. Regional nerve block mitigated the incidence of POD (OR 0.44, 95%CI 0.30 to 0.64; *p* < 0.001; I^2^ = 0.00%) (Figure 2). Sensitivity analysis confirmed that the log OR remained stable, implying that no single study skewed the significance (Figure S1). The analysis revealed no evidence of publication bias, as indicated by both the results of the funnel plot (Figure S2) and Egger’s linear regression test (*p* = 0.693). Additionally, the application of the trim and fill method did not alter the results.

### 3.3. Postoperative Cognitive Dysfunction

**Figure 3:**
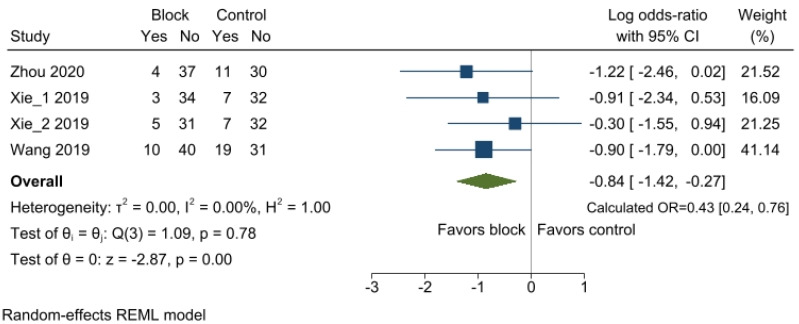
Forest plot of the incidence of POCD between the regional nerve block and control groups. Significant differences were observed in the incidence of POCD in patients who received regional nerve block compared with no block. POCD, postoperative cognitive dysfunction; OR, odds ratio; CI, confidence interval [27,28,29].

The incidence of POCD was reported in three RCTs [27,28,29], and a total of four comparisons were included because two different comparisons were made regarding the incidence of POCD through three groups in one study [28]. In the regional nerve block group, POCD incidence decreased by 57% compared with that in the control group (OR 0.43, 95%CI 0.24 to 0.76; *p* < 0.001; I^2^ = 0.00%) (Figure 3). Sensitivity analysis revealed that the pooled effect size was not changed by omitting the studies (Figure S3). The funnel plot showed symmetry (Figure S4), and Egger’s linear regression test confirmed that no publication bias existed (*p* = 0.915). Furthermore, the trim and fill method did not alter the aggregated findings.

### 3.4. Pain Scores

**Figure 4:**
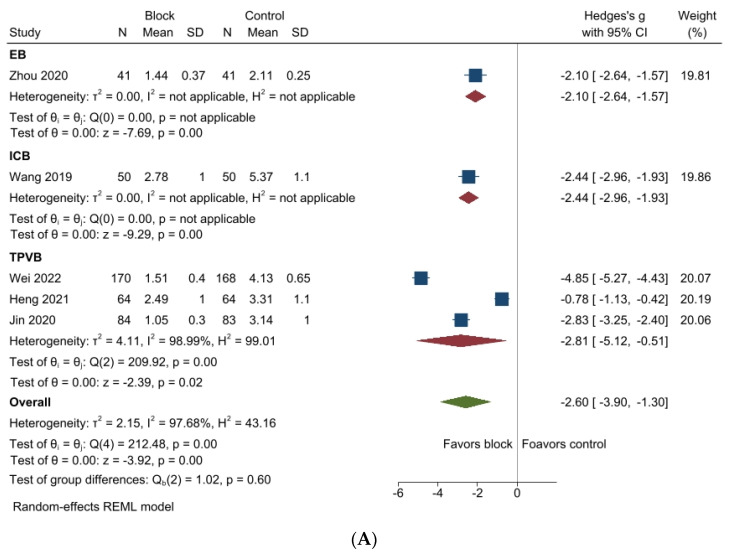

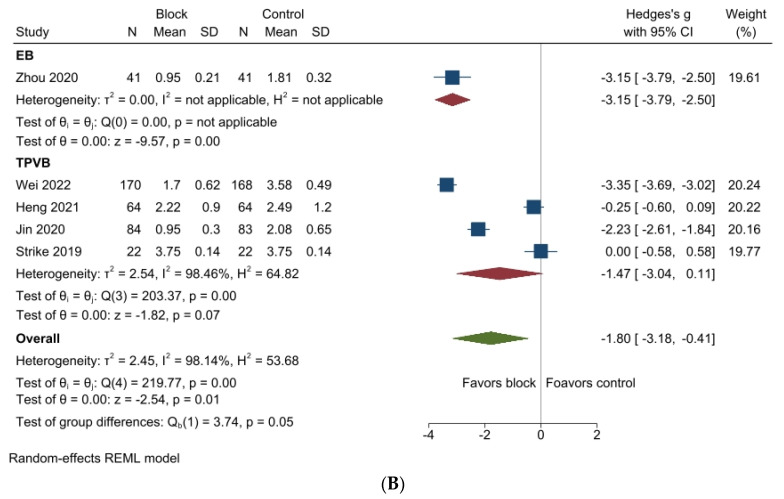
Forest plots for postoperative pain scores between the regional nerve block and control groups. (**A**) Postoperative 24 h and (**B**) postoperative 48 h. Patients in the regional nerve group had significantly lower pain scores at all time points than those in the control group. SD, standard deviation; CI, confidence interval. EB, epidural block; ICB, interscalene nerve block; TPVB, thoracic paravertebral block [8,9,25,26,27,29].

Postoperative pain scores were significantly reduced at both 24 h (SMD −2.60, 95%CI −3.90 to −1.30, *p* < 0.001; I^2^ = 97.68%; Figure 4A) and 48 h (SMD −1.80, 95%CI −3.18 to −0.41, *p* = 0.01; I^2^ = 98.14%; Figure 4B) in the regional nerve block group. In the subgroup analysis, TPVB (SMD −2.81, 95%CI −5.12 to −0.51, *p* = 0.02; Figure 4A), EB (SMD −2.10, 95%CI −2.64 to −1.57, *p* < 0.001; Figure 4A), and ICNB (SMD −2.44, 95%CI −2.93 to −1.93, *p* < 0.01; Figure 4A) significantly decreased the pain scores compared to those of the control group at 24 h postoperatively. Regarding the pain scores 48 h after surgery, while the EB reduced pain scores (SMD −3.15, 95%CI −3.79 to −2.50, *p* < 0.001; Figure 4B) compared with the control group, TPVB did not provide effective analgesia (SMD −1.47, 95%CI −3.04 to 0.11, *p* = 0.07; Figure 4B) compared with the control group. Sensitivity analysis did not reveal any substantial effect size (Figure S5) concerning the 24 h postoperative pain score. Furthermore, the symmetrical funnel plot (Figure S6) and Egger’s linear regression test (*p* = 0.835) confirmed no publication bias for the postoperative 24 h pain score. Furthermore, the trim and fill method neither identified nor added any missing studies. Sensitivity analysis for the postoperative 48 h pain score showed that the effect size changed when some studies were removed [8,26,27] (Figure S7). No publication bias for the postoperative 48 h pain score was confirmed, showing a symmetrical funnel plot (Figure S8) and a non-significant Egger’s test (*p* = 0.960). Additionally, the trim and fill method did not supplement any missing data or alter the results.

Three studies [8,9,26] compared opioid consumption as an outcome, showing no benefit in opioid consumption with NBs compared to the control group (Figure S9). This finding persisted in sensitivity analysis, indicating consistent results (Figure S10). 

## 4. Risk of Bias

The overall risk of bias was assessed as unclear in most of the included studies [9,25,26,27,28,29], and only one study showed a low risk [8], as shown in Figure S11. All patients were randomly assigned to the groups in all included RCTs, but in two studies, the authors did not describe how to generate random sequences [25,29]. Information regarding the selection bias performance was not described in seven studies, which made the risk of bias unclear [9,25,26,27,28,29]. Five [9,25,26,27,29] and two studies [8,28] were judged as unclear and with a low risk of detection bias, respectively. Regarding attrition bias, one study did not explain why some patients were excluded from the final analysis [9]. The risks of reporting and other biases were low in most of the included studies.

## 5. Certainty of Evidence

All outcomes had a high certainty of evidence. Detailed information can be found in Table S3.

## 6. Discussion

This systematic review and meta-analysis identified that regional nerve blocks influence the development of POD and POCD, showing a significant decrease in incidence. In addition, effective analgesia was obtained in the regional nerve group compared to the no-block group. To the best of our knowledge, this is the first systematic review and meta-analysis to report the effects of regional nerve block on postoperative cognitive impairment.

To date, concerns regarding the occurrence of postoperative cognitive impairment have been increasing among healthcare providers. This trend may be due to the risk of various poor outcomes in patients, including prolonged time to return to normal life and the possibility of developing dementia [30,31]. Unfortunately, no definite risk factors have been identified, and various components are known to contribute to postoperative cognitive impairment, making it difficult to prevent or treat POD and POCD.

The precise mechanisms of POD and POCD have not been identified. Among the various etiologies, the role of neuroinflammatory changes has been gaining interest [32]. Furthermore, pain, which could influence the inflammatory process in the brain, is also regarded as a contributing factor to the occurrence of POD or POCD [6,33]. In patients undergoing surgery, inevitable physiologic changes, as described above, occur during the perioperative period. With regard to these issues, a regional nerve block could be a good option for attenuating the inflammatory response and postoperative pain simultaneously.

In this meta-analysis, the incidence of POD and POCD decreased by over 50% when a regional nerve block was performed compared to when no block was performed. Most of the included studies explained the reason for the reduction in POD and POCD based on the anti-neuroinflammatory effect through analgesia of regional nerve blocks, and the evidence was suggested using changes in neuroinflammatory substances, such as interleukin-1β, interleukin-6, tumor necrosis factor-α, and high-sensitivity C-reactive protein [8,25,26,27,28,29]. This opinion is supported by several clinical trials evaluating the role of regional nerve blocks in the inflammatory response after surgery. Bagry et al. [34] reported that continuous lumbar plexus and sciatic nerve block attenuated the inflammatory response after total knee, showing decreased levels of C-reactive protein and leukocyte count. One RCT [35] that evaluated the effect of interscalene nerve block on the inflammatory response demonstrated that nerve blocks may inhibit the progress of inflammation in arthroscopic shoulder surgery. However, caution is required when interpreting the role of regional nerve block in the inflammatory process within the included studies in this meta-analysis. Although the included studies showed a reduced inflammatory reaction by measuring the inflammatory substances, no definite evidence that regional block directly contributed to the anti-inflammatory effects could be found in the analyzed studies. In addition, the association between inflammation and cognitive changes has not been evaluated. Further studies are needed to clarify the anti-inflammatory effect of regional nerve block, the relationship between pain and the inflammatory cascade, and the causality between neuroinflammation and cognitive impairment.

Several studies suggest that sleep deprivation, which can be caused by pain, plays a significant role in the occurrence of POD [36,37], although most studies have focused on patients in intensive care units. Sleep deprivation disrupts the circadian rhythm and decreases the level of melatonin, leading to delirium [38]. Exposure to such sleep deprivation also renders one more sensitive to pain [39]. Studies indicate that within this sequence of events, inflammation, particularly pro-inflammatory cytokines, serve as a mediator [40]. 

Additionally, all patients in the RCTs included in our study underwent thoracic surgeries, which inherently necessitates one lung ventilation throughout the procedure. When undergoing one lung ventilation, hypoxic pulmonary vasoconstriction occurs, leading to the generation of oxidative stress and free radicals, and triggering a pro-inflammatory response [41]. Furthermore, due to surgical stimuli, a peripheral inflammatory response manifests postoperatively [42]. The inflammatory response leads to the destruction of the glycocalyx matrix in vascular endothelial cells, thereby increasing the permeability of the blood–brain barrier [43,44]. As the permeability of the blood–brain barrier increases, it ultimately facilitates greater accessibility of pro-inflammatory cytokines to the central nervous system, contributing to the exacerbation of postoperative cognitive decline [45,46]. 

Complications that may occur after a nerve block include sensory or motor deficits, postoperative nausea or vomiting, itching, hematoma, or abscess formation [47]. Upon closer examination of the nerve blocks utilized in the studies encompassed within our meta-analysis, complications such as 30-day mortality, neurological complications, infection of the catheter or injection site, hematomas, and pruritus were rare when ICNB was performed [10]. Extra caution is necessary with thoracic paravertebral blocks because pneumothorax was reported to occur [48]. When performing an epidural block, there is a risk of subarachnoid block and epidural abscess formation that may lead to potential long-term sequelae. Additionally, complications such as hypotension, pruritus, injection site infections, and post-dural puncture headaches may arise, underscoring the need for careful attention [49].

Several limitations of the present study warrant a careful interpretation of the results. First, various types of nerve block were included in the analysis of POCD incidence. Although the statistical heterogeneity was low, and no small study effect was observed via sensitivity analysis by omitting the studies one by one, clinical heterogeneity seems high. Therefore, it would be necessary to conduct a new meta-analysis after more RCTs targeting similar patient populations and performing similar interventions are published. Second, high heterogeneity was observed in the pain scores. A possible reason for heterogeneity may be the variety of types of surgery, which may cause a different pain intensity according to the surgery postoperatively. In addition, local anesthetics might influence the heterogeneity of pain scores because of the different types, concentrations, and volumes of the local anesthetics used. Third, we did not conduct a trial sequential analysis in our meta-analysis. Trial sequential analysis is a method used in meta-analysis to validate the robustness of findings by reducing type I and type II errors [50]. It is primarily employed in assessing the primary outcome [51]. In our meta-analysis, trial sequential analysis was not conducted for the analysis of the primary outcome, which involves the incidence rates of POD and POCD, as each included study demonstrated consistent effects and directions. However, in the future, if additional RCTs are published and subsequent meta-analyses yield altered results, conducting a trial sequential analysis could be beneficial. This approach would help ensure a more comprehensive and rigorous assessment of the meta-analytical outcomes in light of new evidence from more RCTs.

In conclusion, regional nerve blocks for thoracic surgery via thoracoscopy or thoracotomy reduced the incidence of POD or POCD after surgery and provided effective analgesia. Although regional nerve blocks significantly improved postoperative cognitive impairment, clinical evidence is still lacking because of the limited number of included studies. Further large RCTs are required to strengthen the evidence and confirm our results.

## Figures and Tables

**Table 1:** Characteristics of included trials.

Author (Year)	Group	N (Block/Control)	Nerve Block	Control Arm	Age (Block/Control)	Diagnosis	Diagnostic Tool of Cognitive Function	Types of Surgery	Pain Score	Postoperative Pain Control
Wei (2022) [8]	2	50/50	US-TPVB	No block	76.2/73.5	Delirium	CAM	VATS	VAS	IV-PCA
Heng (2021) [25]	2	64/64	US-TPVB	No block	70.3/69.7	Delirium	NDSS	VATS	VAS	IV-PCA
Jin (2020) [26]	2	90/90	US-TPVB	No block	70.8/71.4	Delirium	CAM	Thoracotomy for esophageal cancer	NRS	IV-PCA
Strike (2019) [9]	2	22/22	US-TPVB	No block	82.3/81.7	Delirium	CAM	Thoracotomy for TAVR	VAS	IV-PCA
Zhou (2020) [27]	2	41/41	EB	No block	56.7/56.3	POCD	MMSE	VATS	VAS	No information
Xie (2019) [28]	3	37/39 36/39	US-TPVB EB	No block No block	75.1/76.6 76.1/76.6	POCD	MMSE	VATS	VAS	IV-PCA IV-PCA
Wang (2019) [29]	2	50/50	ICNB	No block	45.3/44.9	POCD	MMSE	Thoracotomy for esophageal cancer	VAS	No information

US-TPVB, ultrasound-guided thoracic paravertebral block; EB, epidural block; ICNB, intercostal nerve block; CAM, confusion assessment tool; NDSS, nursing delirium screening scale; MMSE, mini-mental state examination; POCD, postoperative cognitive dysfunction; VATS, video-assisted thoracic surgery; TAVR, transapical aortic valve replacement; IV-PCA, intravenous patient-controlled analgesia; VAS, visual analog scale; NRS, numerical rating scale.

**Table 2:** Characteristics of the blocks of included trials.

Author (Year)	Nerve Block	Block Technique	Local Anesthetics	Volume of Local Anesthetics	Injected Adjuncts of Block	Method of Block Confirmation	Postoperative Pain Control
Wei (2022) [8]	US-TPVB	Continuous	0.2% Ropivacaine	2 mL/h	None	Ultrasound-guided	IV-PCA
Heng (2021) [25]	US-TPVB	Continuous	0.5% Ropivacaine	20 mL 2 mL/h	None	Ultrasound-guided	IV-PCA
Jin (2020) [26]	US-TPVB	Continuous	0.375% Ropivacaine	15–20 mL 10 mL/6 h	10 ug of sufentanil	Ultrasound-guided	IV-PCA
Strike (2019) [9]	US-TPVB	Continuous	0.2% Ropivacaine or 0.125% Bupivacaine	5–8 mL 5–10 mL/h	None	Ultrasound-guided	IV-PCA
Zhou (2020) [27]	EB	Continuous	0.75% Ropivacaine	0.12 mL/kg	3 mL of 2% lidocaine	15 min of observation	No information
Xie (2019) [28]	US-TPVB EB	Single shot Single shot	0.25% Ropivacaine 0.375% Ropivacaine	20 mL 8–15 mL	None	Ultrasound-guided	IV-PCA IV-PCA
Wang (2019) [29]	ICNB	Single shot	0.5% Ropivacaine	15 mL	None	No information	No information

US-TPVB, ultrasound-guided thoracic paravertebral block; EB, epidural block; ICNB, intercostal nerve block, IV-PCA, intravenous patient-controlled analgesia.

## Data Availability

No new data were created or analyzed in this study. Data sharing is not applicable to this article.

## Supplementary Materials

The following supporting information can be downloaded at: https://www.mdpi.com/article/10.3390/jcm12247576/s1, Table S1. Search strategy for each database; Table S2. Details for judgement for each risk of bias for randomized controlled studies; Figure S1. Forest plot for sensitivity analysis of the incidence of POD; Figure S2. Funnel plot for the incidence of POD; Figure S3. Forest plot for sensitivity analysis of the incidence of POCD; Figure S4. Funnel plot for the incidence of POCD; Figure S5. Forest plot for sensitivity analysis of postoperative 24 h pain score; Figure S6. Funnel plot for postoperative 24 h pain score; Figure S7. Forest plot for sensitivity analysis of postoperative 48 h pain score; Figure S8. Funnel plot for postoperative 48 h pain score; Figure S9. Forest plot for opioid consumption; Figure S10. Forest plot for sensitivity analysis of opioid consumption; Figure S11. Risk of bias summary. Table S3. Assessments of certainty of evidence for each outcome.

## References

[1] Vacas S., Cole D.J., Cannesson M. (2021). Cognitive Decline Associated With Anesthesia and Surgery in Older Patients. JAMA.

[2] Robinson T.N., Raeburn C.D., Tran Z.V., Angles E.M., Brenner L.A., Moss M. (2009). Postoperative delirium in the elderly: Risk factors and outcomes. Ann. Surg..

[3] Park E.A., Kim M.Y. (2019). Postoperative Delirium is Associated with Negative Outcomes and Long-Term Mortality in Elderly Koreans: A Retrospective Observational Study. Medicina.

[4] Bramley P., McArthur K., Blayney A., McCullagh I. (2021). Risk factors for postoperative delirium: An umbrella review of systematic reviews. Int. J. Surg..

[5] Jin Z., Hu J., Ma D. (2020). Postoperative delirium: Perioperative assessment, risk reduction, and management. Br. J. Anaesth..

[6] Vaurio L.E., Sands L.P., Wang Y., Mullen E.A., Leung J.M. (2006). Postoperative delirium: The importance of pain and pain management. Anesth. Analg..

[7] Jin F., Chung F. (2001). Multimodal analgesia for postoperative pain control. J. Clin. Anesth..

[8] Wei W., Zheng X., Gu Y., Fu W., Tang C., Yao Y. (2022). Effect of general anesthesia with thoracic paravertebral block on postoperative delirium in elderly patients undergoing thoracoscopic lobectomy: A randomized-controlled trial. BMC Anesthesiol..

[9] Strike E., Arklina B., Stradins P., Cusimano R.J., Osten M., Horlick E., Styra R., Poonawala H., Carroll J., Djaiani G. (2019). Postoperative Pain Management Strategies and Delirium After Transapical Aortic Valve Replacement: A Randomized Controlled Trial. J. Cardiothorac. Vasc. Anesth..

[10] Guerra-Londono C.E., Privorotskiy A., Cozowicz C., Hicklen R.S., Memtsoudis S.G., Mariano E.R., Cata J.P. (2021). Assessment of Intercostal Nerve Block Analgesia for Thoracic Surgery: A Systematic Review and Meta-analysis. JAMA Netw. Open.

[11] Jack J., McLellan E., Versyck B., Englesakis M., Chin K. (2020). The role of serratus anterior plane and pectoral nerves blocks in cardiac surgery, thoracic surgery and trauma: A qualitative systematic review. Anaesthesia.

[12] Turhan Ö., Sivrikoz N., Sungur Z., Duman S., Özkan B., Şentürk M. (2021). Thoracic Paravertebral Block Achieves Better Pain Control Than Erector Spinae Plane Block and Intercostal Nerve Block in Thoracoscopic Surgery: A Randomized Study. J. Cardiothorac. Vasc. Anesth..

[13] Khan B.A., Perkins A.J., Campbell N.L., Gao S., Khan S.H., Wang S., Fuchita M., Weber D.J., Zarzaur B.L., Boustani M.A. (2018). Preventing postoperative delirium after major noncardiac thoracic surgery—A randomized clinical trial. J. Am. Geriatr. Soc..

[14] Van Norden J., Spies C., Borchers F., Mertens M., Kurth J., Heidgen J., Pohrt A., Mueller A. (2021). The effect of peri-operative dexmedetomidine on the incidence of postoperative delirium in cardiac and non-cardiac surgical patients: A randomised, double-blind placebo-controlled trial. Anaesthesia.

[15] Shen L., Chen J.-Q., Yang X.-L., Hu J.-C., Gao W., Chai X.-Q., Wang D. (2022). Flurbiprofen used in one-lung ventilation improves intraoperative regional cerebral oxygen saturation and reduces the incidence of postoperative delirium. Front. Psychiatry.

[16] Aiello G., Cuocina M., La Via L., Messina S., Attaguile G.A., Cantarella G., Sanfilippo F., Bernardini R. (2023). Melatonin or Ramelteon for Delirium Prevention in the Intensive Care Unit: A Systematic Review and Meta-Analysis of Randomized Controlled Trials. J. Clin. Med..

[17] Fellous S., Dubost B., Cambriel A., Bonnet M.-P., Verdonk F. (2023). Perioperative ketamine administration to prevent delirium and neurocognitive disorders after surgery: A systematic review and meta-analysis. Int. J. Surg..

[18] Duning T., Ilting-Reuke K., Beckhuis M., Oswald D. (2021). Postoperative delirium—Treatment and prevention. Curr. Opin. Anesthesiol..

[19] Kim S.Y., Jo H.Y., Na H.-S., Han S.-H., Do S.-H., Shin H.-J. (2023). The Effect of Peripheral Nerve Block on Postoperative Delirium in Older Adults Undergoing Hip Surgery: A Systematic Review and Meta-Analysis of Randomized Controlled Trials. J. Clin. Med..

[20] Li T., Dong T., Cui Y., Meng X., Dai Z. (2022). Effect of regional anesthesia on the postoperative delirium: A systematic review and meta-analysis of randomized controlled trials. Front. Surg..

[21] Moher D., Liberati A., Tetzlaff J., Altman D.G., Group P. (2009). Preferred reporting items for systematic reviews and meta-analyses: The PRISMA statement. J. Clin. Epidemiol..

[22] Wan X., Wang W., Liu J., Tong T. (2014). Estimating the sample mean and standard deviation from the sample size, median, range and/or interquartile range. BMC Med. Res. Methodol..

[23] Higgins J.P., Altman D.G., Gotzsche P.C., Juni P., Moher D., Oxman A.D., Savovic J., Schulz K.F., Weeks L., Sterne J.A. (2011). The Cochrane Collaboration’s tool for assessing risk of bias in randomised trials. BMJ.

[24] Shim J.G., Ryu K.H., Kim P.O., Cho E.A., Ahn J.H., Yeon J.E., Lee S.H., Kang D.Y. (2020). Evaluation of ultrasound-guided erector spinae plane block for postoperative management of video-assisted thoracoscopic surgery: A prospective, randomized, controlled clinical trial. J. Thorac. Dis..

[25] Heng L., Wang M., Wang M., Li L., Zhu S. (2021). Thoracic Paravertebral Block Ameliorates Postoperative Delirium in Geriatric Patients. Thorac. Cardiovasc. Surg..

[26] Jin L., Yao R., Heng L., Pang B., Sun F.G., Shen Y., Zhong J.F., Zhao P.P., Wu C.Y., Li B.P. (2020). Ultrasound-guided continuous thoracic paravertebral block alleviates postoperative delirium in elderly patients undergoing esophagectomy: A randomized controlled trial. Medicine.

[27] Zhou W., Wang B., Cai X., Xu Z. (2020). Effects of epidural block anesthesia combined with general anesthesia on cognitive function and analgesic effect after thoracoscopic surgery. Int. J. Clin. Exp. Med..

[28] Xie H., Zhou J., Du W., Zhang S., Huang R., Han Q., Guo Q. (2019). Impact of thoracic paravertebral block combined with general anesthesia on postoperative cognitive function and serum adiponectin levels in elderly patients undergoing lobectomy. Videosurg. Other Miniinvasive Tech..

[29] Wang Y., Cheng J., Yang L., Wang J., Liu H., Lv Z. (2019). Ropivacaine for Intercostal Nerve Block Improves Early Postoperative Cognitive Dysfunction in Patients Following Thoracotomy for Esophageal Cancer. Med. Sci. Monit..

[30] Silverstein J.H., Deiner S.G. (2013). Perioperative delirium and its relationship to dementia. Prog. Neuropsychopharmacol. Biol. Psychiatry.

[31] Liang C.K., Chu C.L., Chou M.Y., Lin Y.T., Lu T., Hsu C.J., Chen L.K. (2014). Interrelationship of postoperative delirium and cognitive impairment and their impact on the functional status in older patients undergoing orthopaedic surgery: A prospective cohort study. PLoS ONE.

[32] Cerejeira J., Firmino H., Vaz-Serra A., Mukaetova-Ladinska E.B. (2010). The neuroinflammatory hypothesis of delirium. Acta Neuropathol..

[33] Campos A.C.P., Antunes G.F., Matsumoto M., Pagano R.L., Martinez R.C.R. (2020). Neuroinflammation, Pain and Depression: An Overview of the Main Findings. Front. Psychol..

[34] Bagry H., de la Cuadra Fontaine J.C., Asenjo J.F., Bracco D., Carli F. (2008). Effect of a continuous peripheral nerve block on the inflammatory response in knee arthroplasty. Reg. Anesth. Pain. Med..

[35] Mejia-Terrazas G.E., Ruiz-Suarez M., Vadillo-Ortega F., Franco Y.B.R.E., Lopez-Munoz E. (2019). Effect of interscalene nerve block on the inflammatory response in shoulder surgery: A randomized trial. J. Shoulder Elb. Surg..

[36] Wang H., Zhang L., Zhang Z., Li Y., Luo Q., Yuan S., Yan F. (2020). Perioperative Sleep Disturbances and Postoperative Delirium in Adult Patients: A Systematic Review and Meta-Analysis of Clinical Trials. Front. Psychiatry.

[37] He E., Dong Y., Jia H., Yu L. (2022). Relationship of sleep disturbance and postoperative delirium: A systematic review and meta-analysis. Gland. Surg..

[38] Olofsson K., Alling C., Lundberg D., Malmros C. (2004). Abolished circadian rhythm of melatonin secretion in sedated and artificially ventilated intensive care patients. Acta Anaesthesiol. Scand..

[39] Herrero Babiloni A., De Koninck B.P., Beetz G., De Beaumont L., Martel M.O., Lavigne G.J. (2020). Sleep and pain: Recent insights, mechanisms, and future directions in the investigation of this relationship. J. Neural Transm..

[40] Yang D.-F., Huang W.-C., Wu C.W., Huang C.-Y., Yang Y.-C.S.H., Tung Y.-T. (2023). Acute sleep deprivation exacerbates systemic inflammation and psychiatry disorders through gut microbiota dysbiosis and disruption of circadian rhythms. Microbiol. Res..

[41] van der Woude M.C., Bormans L., van der Horst R.P., Sosef M.N., Belgers H.J., Hemmes S.N., Tuip-de Boer A., de Abreu M.G., Pelosi P., Spronk P.E. (2020). Pulmonary levels of biomarkers for inflammation and lung injury in protective versus conventional one-lung ventilation for oesophagectomy: A randomised clinical trial. Eur. J. Anaesthesiol..

[42] Verdonk F., Einhaus J., Tsai A.S., Hedou J., Choisy B., Gaudilliere D., Kin C., Aghaeepour N., Angst M.S., Gaudilliere B. (2021). Measuring the human immune response to surgery: Multiomics for the prediction of postoperative outcomes. Curr. Opin. Crit. Care.

[43] Sommer C., Leinders M., Üçeyler N. (2018). Inflammation in the pathophysiology of neuropathic pain. Pain.

[44] Rahbar E., Cardenas J.C., Baimukanova G., Usadi B., Bruhn R., Pati S., Ostrowski S.R., Johansson P.I., Holcomb J.B., Wade C.E. (2015). Endothelial glycocalyx shedding and vascular permeability in severely injured trauma patients. J. Transl. Med..

[45] Wang J.-Y., Li M., Wang P., Fang P. (2022). Goal-directed therapy based on rScO2 monitoring in elderly patients with one-lung ventilation: A randomized trial on perioperative inflammation and postoperative delirium. Trials.

[46] Devinney M.J., Wong M.K., Wright M.C., Marcantonio E.R., Terrando N., Browndyke J.N., Whitson H.E., Cohen H.J., Nackley A.G., Klein M.E. (2023). Role of Blood–Brain Barrier Dysfunction in Delirium following Non-cardiac Surgery in Older Adults. Ann. Neurol..

[47] Campos M.G., Peixoto A.R., Fonseca S., Santos F., Pinho C., Leite D. (2022). Assessment of main complications of regional anesthesia recorded in an acute pain unit in a tertiary care university hospital: A retrospective cohort. Braz. J. Anesthesiol..

[48] Niesen A.D., Jacob A.K., Law L.A., Sviggum H.P., Johnson R.L. (2020). Complication rate of ultrasound-guided paravertebral block for breast surgery. Reg. Anesth. Pain. Med..

[49] Manassero A., Bossolasco M., Carrega M., Coletta G. (2020). Postoperative Thoracic Epidural Analgesia: Adverse Events from a Single-Center Series of 3126 Patients. Local Reg. Anesth..

[50] Sanfilippo F., La Via L., Tigano S., Morgana A., Rosa V., Astuto M. (2021). Trial Sequential Analysis: The evaluation of the robustness of meta-analyses findings and the need for further research. EuroMediterranean Biomed. J..

[51] De Cassai A., Pasin L., Boscolo A., Salvagno M., Navalesi P. (2020). Trial sequential analysis: Plain and simple. Korean J. Anesthesiol..

