# Supplementary material for: The Impact of Regional Nerve Blocks on Postoperative Delirium or Cognitive Dysfunction following Thoracic Surgery: A Systematic Review and Meta-Analysis
Source: J Clin Med. 2023 Dec 8;12(24):7576. doi: 10.3390/jcm12247576 (PMC10743822; doi:10.3390/jcm12247576)
Supplement: Supplementary file 1 [file jcm-12-07576-s001.zip › jcm-2719342-supplementary.pdf]

Supplementary Files

Table S1. Search strategy for each database.

Database	Order	Keywords	Results
PubMed	#1	block[TIAB] OR "nerve block"[MH] OR “nerve block*”[MH] OR “nerve block”[TIAB] OR “nerve block*”[TIAB] OR PVB[TIAB]	263240
	#2	delirium[MH] OR delirium[TIAB] OR “cognitive dysfunction”[MH] OR “cognitive dysfunction”[TIAB] OR “cognitive impairment”[TIAB] OR cognition[MH] OR cognition[TIAB]	327121
	#3	thoracic[TIAB] OR thoracotomy[MH] OR thoracotomy[TIAB] OR thoracotom*[TIAB] OR thorac*[TIAB] OR thoracoscopy[MH] OR thoracoscopy[TIAB] OR thoracoscopic[TIAB]	217469
	#4	#2 AND #3	496
	#5	#1 AND #4	20
EMBASE	#1	(block OR block:ab,ti OR 'nerve block':ab,ti OR 'nerve block*':ab,ti OR pvb:ab,ti) AND ([controlled clinical trial]/lim OR [randomized controlled trial]/lim) AND ([article]/lim OR [article in press]/lim) AND [english]/lim AND [embase]/lim	14365
	#2	('delirium'/exp OR delirium:ab,ti OR 'cognitive dysfunction':ab,ti OR 'cognitive impairment':ab,ti OR cognition:ab,ti) AND ([controlled clinical trial]/lim OR [randomized controlled trial]/lim) AND ([article]/lim OR [article in press]/lim) AND [english]/lim AND [humans]/lim AND [embase]/lim	7882
	#3	(thoracic:ab,ti OR 'thoracotomy'/exp OR thoracotomy:ab,ti OR thorac*:ab,ti OR thoracoscopy:ab,ti OR thoracoscopic:ab,ti) AND ([controlled clinical trial]/lim OR [randomized controlled trial]/lim) AND ([article]/lim OR [article in press]/lim) AND [english]/lim AND [humans]/lim AND [embase]/lim	6005
	#4	#2 AND #3	66
	#5	#1 AND #4	10
SCOPUS	#1	(TITLE-ABS-KEY (block) OR TITLE-ABS-KEY ('nerve AND block') OR TITLE-ABS-KEY ('nerve AND block*') OR TITLE-ABS-KEY (pvb)) AND (LIMIT-TO (DOCTYPE , "ar")) AND (LIMIT-TO (SRCTYPE , "j")) AND (LIMIT-TO (SUBJAREA , "MEDI"))	171029
	#2	(TITLE-ABS-KEY (delirium) OR TITLE-ABS-KEY ('cognitive AND dysfunction') OR TITLE-ABS-KEY ('cognitive AND impairment') OR TITLE-ABS-KEY (cognition) OR TITLE-ABS-KEY (cognitive)) AND (LIMIT-TO (DOCTYPE , "ar")) AND (LIMIT-TO (SUBJAREA , "MEDI")) AND (LIMIT-TO (SRCTYPE , "j"))	303877
	#3	(TITLE-ABS-KEY (thoracic) OR TITLE-ABS-KEY (thoracotomy) OR TITLE-ABS-KEY (thoracotom*) OR TITLE-ABS-KEY (thorac*) OR TITLE-ABS-KEY (thoracoscopy) OR TITLE-ABS-KEY (thoracoscopic)) AND (LIMIT-TO (DOCTYPE , "ar")) AND (LIMIT-TO (SUBJAREA , "MEDI")) AND (LIMIT-TO (SRCTYPE , "j"))	222593
	#4	#2 AND #3	1162
	#5	#1 AND #4	38

CINAHL	S1	MH block OR TI block OR AB block OR MH 'nerve block" OR TI 'nerve block" OR AB 'nerve block" OR MH 'nerve block*' OR TI 'nerve block*' OR AB 'nerve block*'	16051
	S2	MH delirium OR TI delirium OR AB delirium OR MH 'cognitive dysfunction' OR TI 'cognitive dysfunction' OR AB 'cognitive dysfunction' OR MH 'cognitive impairment' OR TI 'cognitive impairment' OR AB 'cognitive impairment'	11769
	S3	MH thoracic OR TI thoracic OR AB thoracic OR MH thoracotomy OR TI thoracotomy OR AB thoracotomy OR MH thorac* OR TI thorac* OR AB thorac* OR MH thoracosco* OR TI thoracosco* OR AB thoracosco*	14231
	S4	S2 AND S3	29
	S5	S1 AND S4	3
Web of Science	#1	block (Topic) or block (Title) or block (Abstract) or 'nerve block' (Topic) or 'nerve block' (Title) or 'nerve block' (Abstract) or block* (Topic) or block* (Title) or block* (Abstract) and Articles (Document Types)	1026163
	#2	delirium (Topic) or delirium (Title) or delirium (Abstract) or 'cognitive dysfunction' (Topic) or 'cognitive dysfunction' (Title) or 'cognitive dysfunction' (Abstract) or 'cognitive impairment' (Topic) or 'cognitive impairment' (Title) or 'cognitive impairment' (Abstract) and Review Articles (Exclude – Document Types) and Meeting Abstracts or Editorial Materials or Letters (Exclude – Document Types) and Articles (Document Types)	149206
	#3	thoracic (Topic) or thoracic (Title) or thoracic (Abstract) or thoracotomy (Topic) or thoracotomy (Title) or thoracotomy (Abstract) or thoracotom* (Topic) or thoracotom* (Title) or thoracotom* (Abstract) or thoracoscopy (Topic) and thoracoscopy (Title) and thoracoscopy (Abstract) and thoracoscopy (Topic) and thoracoscopy (Title) and thoracoscopy (Abstract) and Review Articles (Exclude – Document Types) and Articles (Document Types)	127277
	#4	#2 AND #3	355
	#5	#1 AND #4	25

Table S2. Details for judgement for each risk of bias for randomized controlled studies.

Study	Bias	Author's judgement	Reason for judgement
Wei 2022 [8]	Random sequence generation (selection bias)	Low	Randomized treatment assignments with a 1:1 ratio were computer generated based on block randomization with random block sizes
	Allocation concealment (selection bias)	Low	Sealed in sequentially numbered envelops and stored by primary investigator
	Blinding (performance)	Low	The investigators responsible for postoperative follow up and the statisticians were all blinded to the randomization until the final statistical analyses were completed.
	Blinding (detection bias)	Low	The investigators responsible for postoperative follow up and the statisticians were all blinded to the randomization until the final statistical analyses were completed.
	Incomplete outcome data (attrition bias)	Low	Outcomes were reported for all patients.
	Selective reporting (reporting bias)	Low	All pre-specified and expected outcomes are reported.
	Other bias	Low	No other bias was detected.
Heng 2021 [25]	Random sequence generation (selection bias)	Unclear	The method was not described in the manuscript.
	Allocation concealment (selection bias)	Unclear	It was not described in the manuscript
	Blinding (performance)	Unclear	It was not described in the manuscript
	Blinding (detection bias)	Unclear	It was not described in the manuscript
	Incomplete outcome data (attrition bias)	Low	Some patients in both groups were excluded from the final analysis with acceptable reasons.
	Selective reporting (reporting bias)	Low	All pre-specified and expected outcomes are reported.
	Other bias	Low	No other bias was detected.
Jin 2020 [26]	Random sequence generation (selection bias)	Low	The randomization was performed using an online randomization tool (http://www.randomization.com).
	Allocation concealment (selection bias)	Unclear	It was not described in the manuscript

	Blinding (performance)	Unclear	It was not described in the manuscript
	Blinding (detection bias)	Unclear	It was not described in the manuscript
	Incomplete outcome data (attrition bias)	Low	Some patients in both groups were excluded from the final analysis with acceptable reasons.
	Selective reporting (reporting bias)	Low	All pre-specified and expected outcomes are reported.
	Other bias	Low	No other bias was detected.
Strike 2019 [9]	Random sequence generation (selection bias)	Low	Participants were randomly assigned in a 1:1 ratio to either the paravertebral group or the PCA group using a computer-generated randomization code in blocks 4.
	Allocation concealment (selection bias)	Unclear	It was not described in the manuscript
	Blinding (performance)	Unclear	It was not described in the manuscript
	Blinding (detection bias)	Unclear	It was not described in the manuscript
	Incomplete outcome data (attrition bias)	Unclear	No reasons were provided why some patients were excluded from the final analysis.
	Selective reporting (reporting bias)	Low	All pre-specified and expected outcomes are reported.
	Other bias	Low	No other bias was detected.
Zhou 2020 [27]	Random sequence generation (selection bias)	Low	According to a random number method
	Allocation concealment (selection bias)	Unclear	It was not described in the manuscript
	Blinding (performance)	Unclear	It was not described in the manuscript
	Blinding (detection bias)	Unclear	It was not described in the manuscript
	Incomplete outcome data (attrition bias)	Low	Outcomes were reported for all patients.
	Selective reporting (reporting bias)	Low	All pre-specified and expected outcomes are reported.
	Other bias	Low	No other bias was detected.
Xie 2019 [28]	Random sequence generation (selection bias)	Low	Using a randomized digital table method
	Allocation concealment (selection bias)	Unclear	It was not described in the manuscript
	Blinding (performance)	Unclear	It was not described in the manuscript
	Blinding (detection bias)	Low	Performed by a researcher blinded to the

			grouping allocation
	Incomplete outcome data (attrition bias)	Low	Eight patients were excluded due to acceptable reason.
	Selective reporting (reporting bias)	Low	All pre-specified and expected outcomes are reported.
	Other bias	Low	No other bias was detected.
Wang 2019 [29]	Random sequence generation (selection bias)	Unclear	It was not described in the manuscript
	Allocation concealment (selection bias)	Unclear	It was not described in the manuscript
	Blinding (performance)	Unclear	It was not described in the manuscript
	Blinding (detection bias)	Unclear	It was not described in the manuscript
	Incomplete outcome data (attrition bias)	Low	Outcomes were reported for all patients.
	Selective reporting (reporting bias)	Low	All pre-specified and expected outcomes are reported.
	Other bias	Low	No other bias was detected.

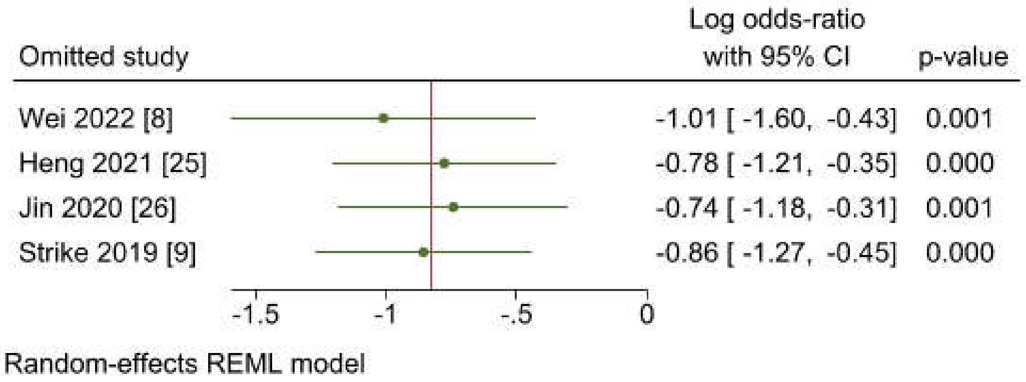

Figure S1. Forest plot for sensitivity analysis of the incidence of POD. Sensitivity analysis confirmed that log odds-ratio remained stable, implying that any single study did not skew the significance. POD, postoperative delirium; CI, confidence interval.

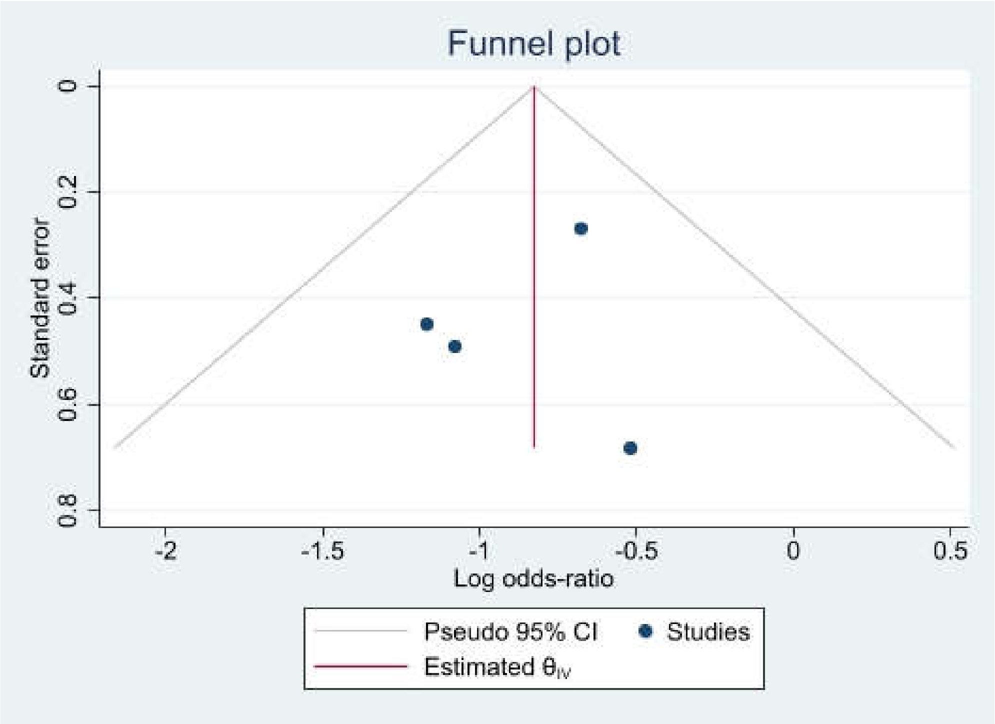

Figure S2. Funnel plot for the incidence of POD.

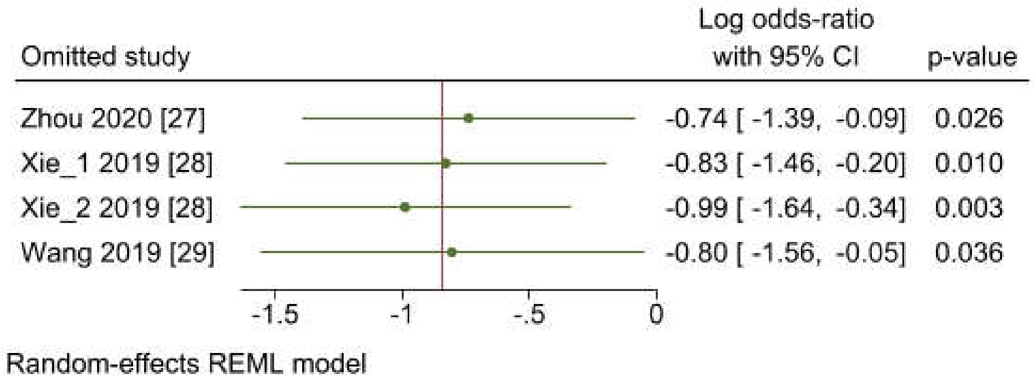

Figure S3. Forest plot for sensitivity analysis of the incidence of POCD. Sensitivity analysis identified that pooled effect size was not changed by omitting studies. POCD, postoperative cognitive dysfunction; CI, confidence interval.

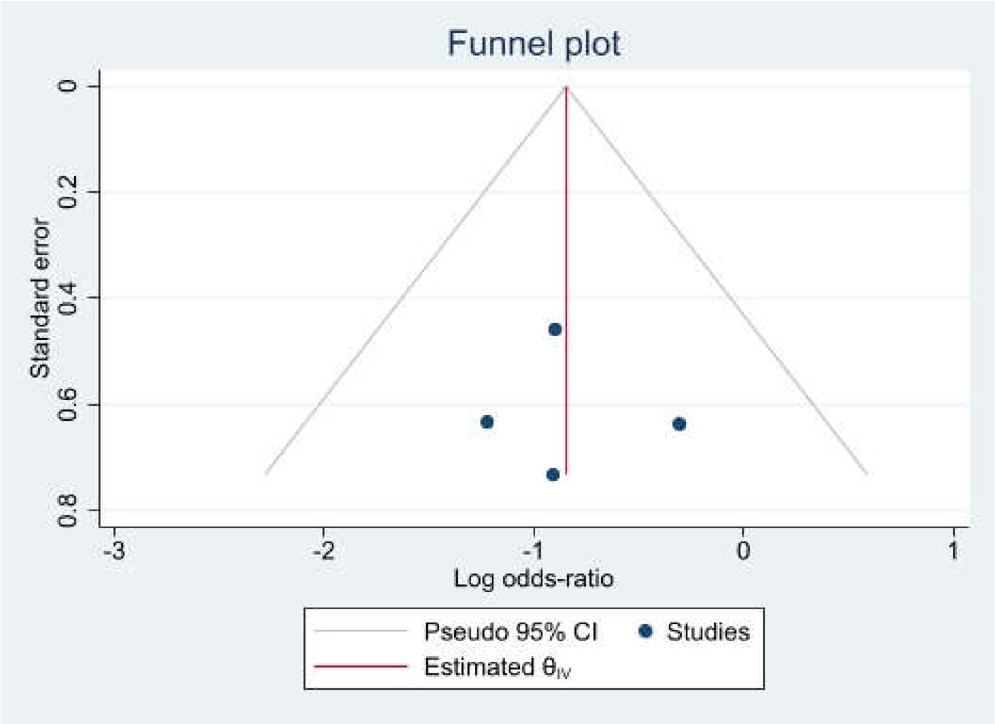

Figure S4. Funnel plot for the incidence of POCD. POCD, postoperative cognitive dysfunction; CI, confidence interval.

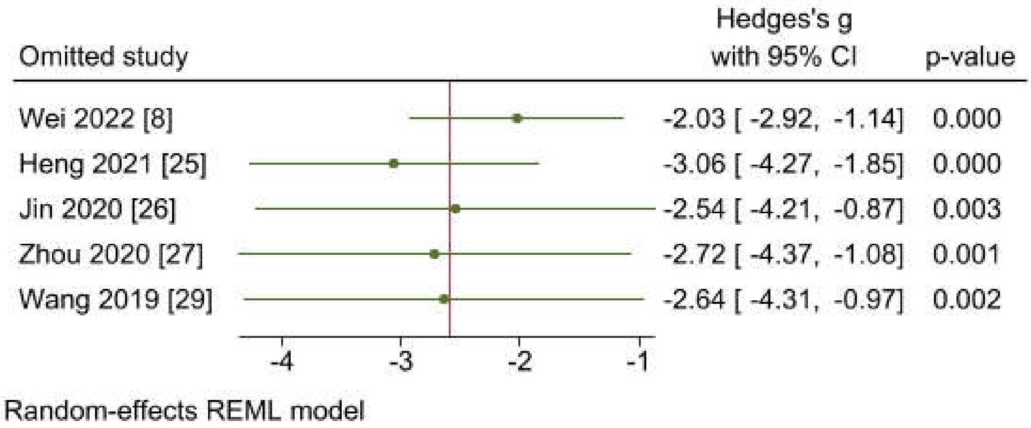

Figure S5. Forest plot for sensitivity analysis of postoperative 24 h pain score. Sensitivity analysis did not detect a meaningful alteration in effect size at 24 h postoperative pain score. CI, confidence interval.

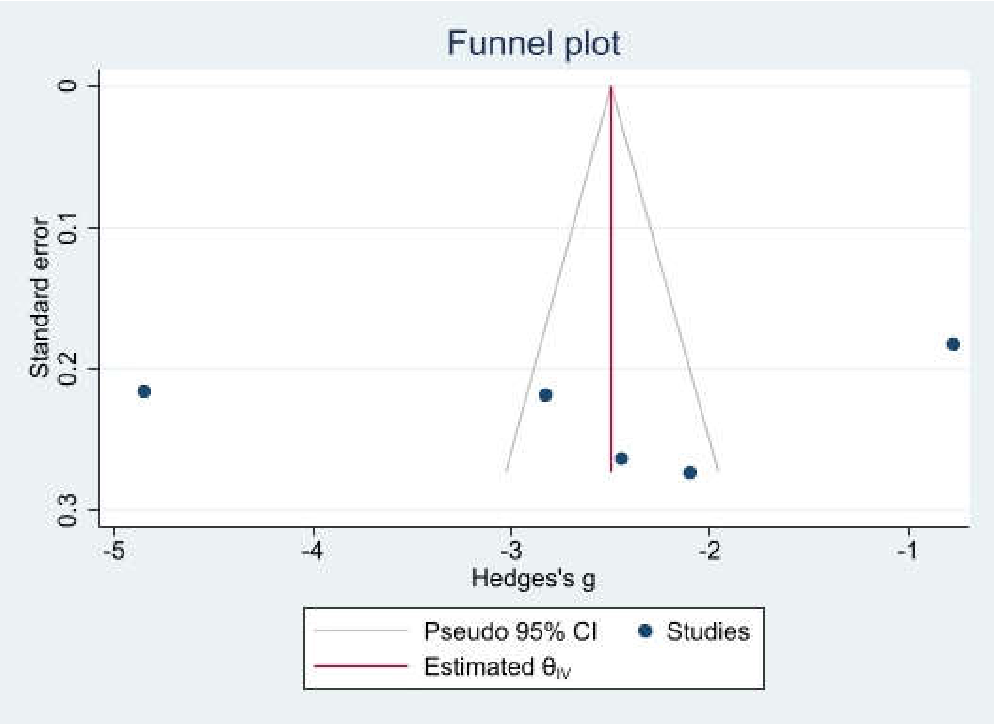

Figure S6. Funnel plot for postoperative 24 h pain score.

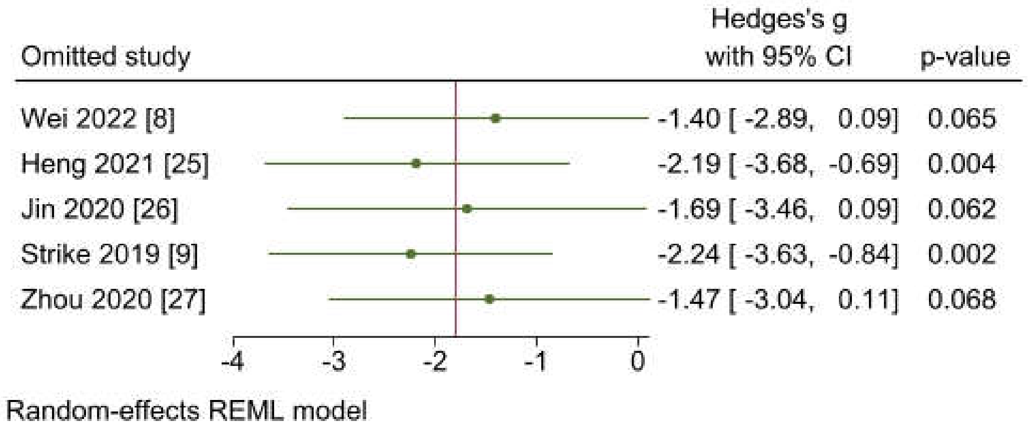

Figure S7. Forest plot for sensitivity analysis of postoperative 48 h pain score. Sensitivity analysis for postoperative 48 h pain score showed effect size changes when some studies were removed [6, 12, 13]. CI, confidence interval.

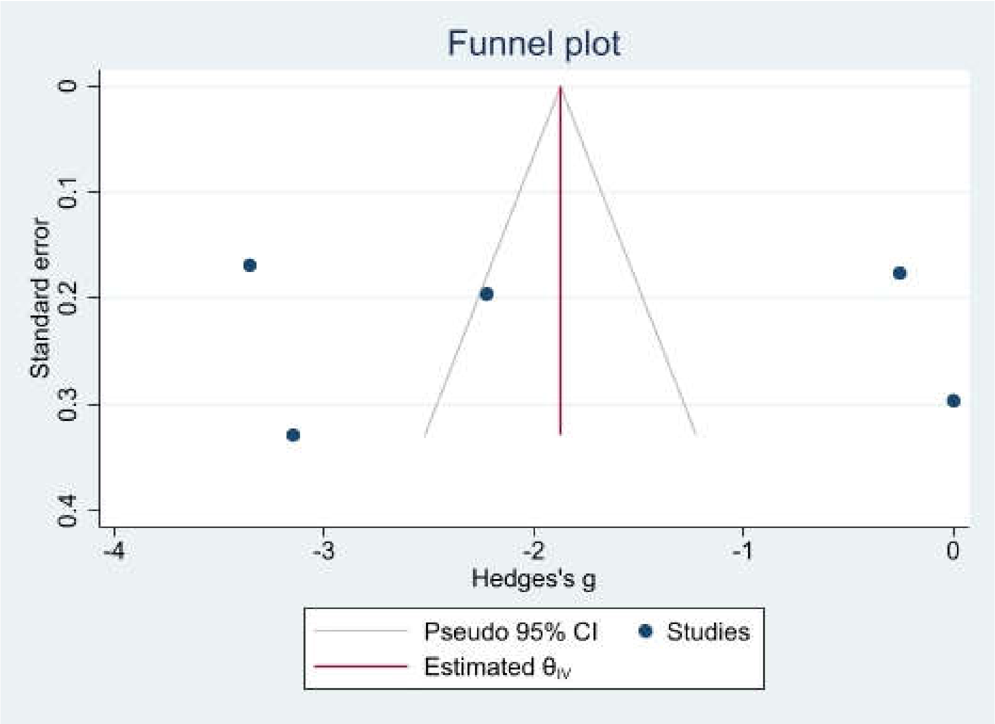

Figure S8. Funnel plot for postoperative 48 h pain score

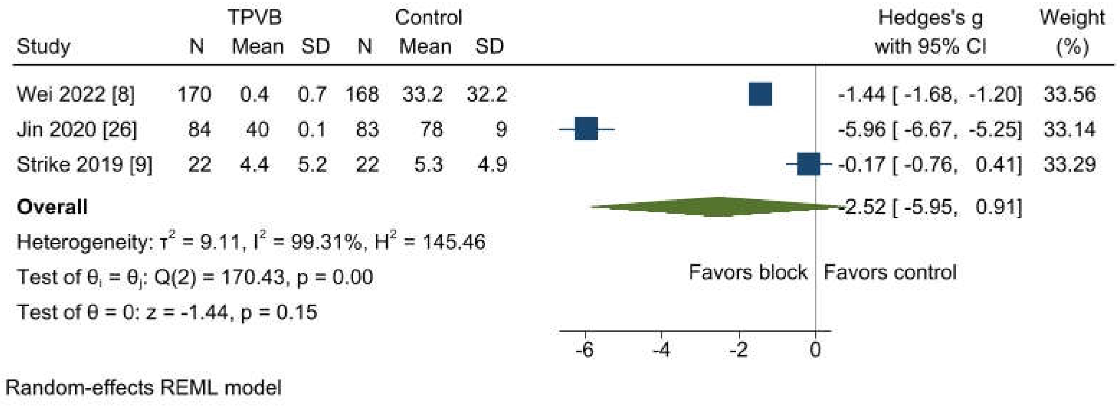

Figure S9. Forest plot for opioid consumption. TPVB, thoracic paravertebral block; SD, standard deviation; CI, confidence interval.

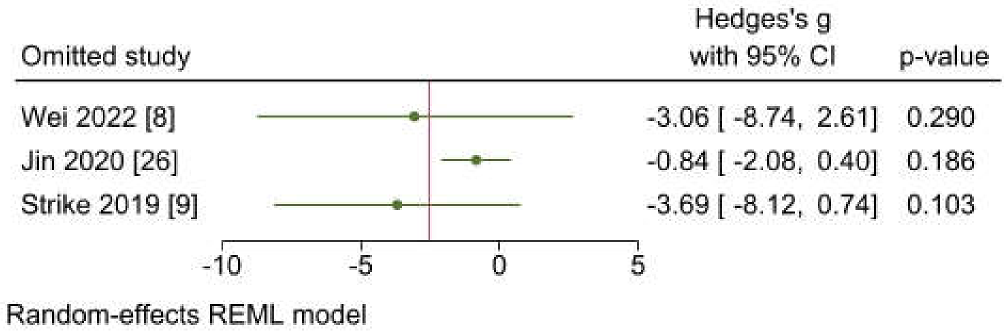

Figure S10. Forest plot for sensitivity analysis of opioid consumption. Sensitivity analysis revealed no substantial change in effect size concerning opioid consumption. CI, confidence interval.

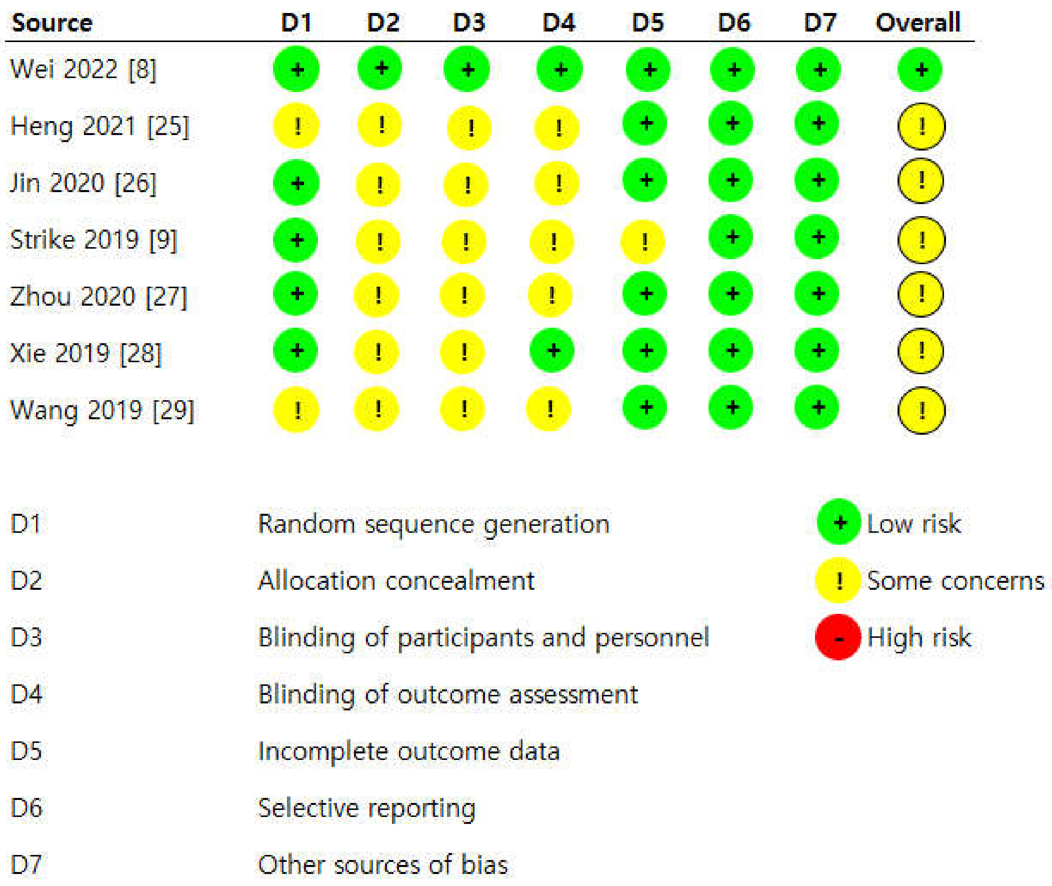

Figure S11. Risk of bias summary. Green circle, low risk; yellow circle, unclear; red circle, high risk

Table S3. Assessments of certainty of evidence for each outcome.

Certainty assessment							№ of patients		Effect		Certainty	Importance
№ of studies	Study design	Risk of bias	Inconsistency	Indirectness	Imprecision	Other considerations	nerve block	no block	Relative (95% CI)	Absolute (95% CI)		
**POD**												
4	randomised trials	not serious	not serious	not serious	not serious	none	48/341 (14.1%)	92/337 (27.3%)	**OR 0.44** (0.30 to 0.64)	**131 fewer per 1,000** (from 172 fewer to 79 fewer)	⨁⨁⨁⨁ High	
**POCD**												
4	randomised trials	not serious	not serious	not serious	not serious	none	22/164 (13.4%)	44/169 (26.0%)	**OR 0.43** (0.24 to 0.76)	**129 fewer per 1,000** (from 182 fewer to 49 fewer)	⨁⨁⨁⨁ High	
**Pain scores at postoperative 24 h (Scale from: 0 to 10)**												
5	randomised trials	not serious	not serious	not serious	not serious	none	409	406	-	SMD **2.6 SD lower** (3.9 lower to 1.3 lower)	⨁⨁⨁⨁ High	
**Pain scores at postoperative 48 h (Scale from: 0 to 10)**												

Certainty assessment							№ of patients		Effect		Certainty	Importance
№ of studies	Study design	Risk of bias	Inconsistency	Indirectness	Imprecision	Other considerations	nerve block	no block	Relative (95% CI)	Absolute (95% CI)		
5	randomised trials	not serious	not serious	not serious	not serious	none	381	378	-	SMD **1.8 SD lower** (3.18 lower to 0.41 lower)	⨁⨁⨁⨁ High	

CI: confidence interval; **OR:** odds ratio; **SMD:** standardised mean difference
